# Biopharmaceutical Assessment of Mesh Aerosolised Plasminogen, a Step towards ARDS Treatment

**DOI:** 10.3390/pharmaceutics15061618

**Published:** 2023-05-30

**Authors:** Lucia Vizzoni, Chiara Migone, Brunella Grassiri, Ylenia Zambito, Baldassare Ferro, Paolo Roncucci, Filippo Mori, Alfonso Salvatore, Ester Ascione, Roberto Crea, Semih Esin, Giovanna Batoni, Anna Maria Piras

**Affiliations:** 1 Department of Pharmacy, University of Pisa, 56126 Pisa, Italy; 2 Department of Life Sciences, University of Siena, 53100 Siena, Italy; 3 Research Centre for Nutraceutical and Healthy Foods “NUTRAFOOD”, University of Pisa, 56124 Pisa, Italy; 4 Anestesia e Rianimazione, Azienda USL Toscana Nord Ovest, 57124 Livorno, Italy; 5 Kedrion S.p.A., Via di Fondovalle, Loc. Bolognana, 55027 Gallicano, Italy; 6 Department of Translational Research and New Technologies in Medicine and Surgery, University of Pisa, 56126 Pisa, Italy; 7 Centre for Instrument Sharing of University of Pisa (CISUP), 56126 Pisa, Italy

**Keywords:** ARDS, COVID-19, SARS-CoV-2, C-ARDS, plasminogen, pulmonary delivery, lung in vitro model, immunomodulating activity

## Abstract

Acute respiratory distress syndrome (ARDS) is a severe complication of lung injuries, commonly associated with bacterial, fungal and viral infections, including SARS-CoV-2 viral infections. ARDS is strongly correlated with patient mortality and its clinical management is very complex, with no effective treatment presently available. ARDS involves severe respiratory failure, fibrin deposition in both airways and lung parenchyma, with the development of an obstructing hyaline membrane drastically limiting gas exchange. Moreover, hypercoagulation is related to deep lung inflammation, and a pharmacological action toward both aspects is expected to be beneficial. Plasminogen (PLG) is a main component of the fibrinolytic system playing key roles in various inflammation regulatory processes. The inhalation of PLG has been proposed in the form of the off-label administration of an eyedrop solution, namely, a plasminogen-based orphan medicinal product (PLG-OMP), by means of jet nebulisation. Being a protein, PLG is susceptible to partial inactivation under jet nebulisation. The aim of the present work is to demonstrate the efficacy of the mesh nebulisation of PLG-OMP in an in vitro simulation of clinical off-label administration, considering both the enzymatic and immunomodulating activities of PLG. Biopharmaceutical aspects are also investigated to corroborate the feasibility of PLG-OMP administration by inhalation. The nebulisation of the solution was performed using an Aerogen^®^ Solo^TM^ vibrating-mesh nebuliser. Aerosolised PLG showed an optimal in vitro deposition profile, with 90% of the active ingredient impacting the lower portions of a glass impinger. The nebulised PLG remained in its monomeric form, with no alteration of glycoform composition and 94% of enzymatic activity maintenance. Activity loss was observed only when PLG-OMP nebulisation was performed under simulated clinical oxygen administration. In vitro investigations evidenced good penetration of aerosolised PLG through artificial airway mucus, as well as poor permeation across an Air–Liquid Interface model of pulmonary epithelium. The results suggest a good safety profile of inhalable PLG, excluding high systemic absorption but with good mucus diffusion. Most importantly, the aerosolised PLG was capable of reversing the effects of an LPS-activated macrophage RAW 264.7 cell line, demonstrating the immunomodulating activity of PLG in an already induced inflammatory state. All physical, biochemical and biopharmaceutical assessments of mesh aerosolised PLG-OMP provided evidence for its potential off-label administration as a treatment for ARDS patients.

## 1. Introduction

Acute respiratory distress syndrome (ARDS) is described as noncardiogenic pulmonary oedema accompanied by severe lung inflammation, hypoxemia and decreased lung compliance. Respiratory failure is therefore the consequence of reduced alveolar–capillary oxygen transfer due to inflammation, vascular microthrombus and hyaline membrane/oedema in the alveoli [1,2].

For ARDS, there is no established gold standard for diagnosis, and it is still unknown whether the condition is caused by a single pathophysiologic mechanism or a number of processes with comparable clinical manifestations. As reported in the ATS/ERS criteria, from a histological point of view, ARDS is characterised by neutrophil infiltration, fluid in the alveoli and the presence of microthrombus, i.e., the triad of ARDS [3]. Increased pulmonary microvascular permeability can be brought about by several direct and indirect lung injuries, allowing protein-rich fluid to enter the alveolar spaces of the lung at normal hydrostatic pressures.

ARDS is a severe complication of lung injuries such as pneumonia, arising from either viral, fungus or bacterial infection. Furthermore, whatever the initial lung injury, patients with ARDS are greatly susceptible to secondary pulmonary infection [4,5]. Despite the improvement in our understanding of ARDS pathophysiology and treatment, the patient mortality rate remains extremely high (35–40%) [6]. ARDS can also result from SARS-CoV-2 infection, which mainly causes endothelial damages. Nearly 75% of COVID-19 patients admitted to intensive care units have thrombotic coagulopathy, and both the clinical picture and the pathologic results are consistent with occlusive microvasculature. When ARDS occurs as part of COVID-19, it is called COVID-19-related acute respiratory distress syndrome (C-ARDS). This new type of severe acute respiratory syndrome meets the Berlin 2012 ARDS definition, even if it seems to be a disease with different phenotypes [7]. The main distinction between C-ARDS and traditional ARDS is the preservation of the patients’ respiratory mechanics in the face of severe hypoxemia. Indeed, the severity of ARDS is significantly associated with the mortality rate of critically ill patients [8], and recently [9], the significant correlation between hypercoagulation and inflammation (neutrophil infiltration and activation) was confirmed.

The main goal for treating ARDS is improved oxygenation and ventilation, often combining adjunctive (neuromuscular blocking agents, prone positioning) and pharmacological therapies [10]. As part of the therapy for the underlying disease (such as shock, trauma, sepsis, pneumonia, aspiration or burns), mechanical ventilation is critical for resolving life-threatening hypoxia and needs careful monitoring to avoid overinflation, barotrauma and cyclic closing/reopening of the alveoli [11]. Despite several clinical trials, few and often controversial positive results have been obtained. Salbutamol (conventionally applied for asthma and chronic obstructive pulmonary disease (COPD)) has shown poor tolerability and has been found to worsen ARDS patient outcomes [12]; simvastatin (an HMG-CoA reductase inhibitor) has shown no effects on ARDS outcomes [13]; and Interferon β-1a (inhibits tumour necrosis factor (TNF), exerts antiviral and antiproliferative properties) has led to no significant reduction in ARDS patient mortality [14]. For C-ARDS patients, only Favipiravir (anti-viral agent, inhibits the RNA-dependent RNA polymerase of RNA viruses) and Tocilizumab (humanised anti-human IL-6 receptor antibody) have shown a significant association with the course of severity of ARDS [15,16,17]. In contrast, the effectiveness of corticosteroids in viral ARDS remains controversial [18]. This scenario evidences an urgent need for a pharmacologically effective ARDS treatment. As such, the off-label administration of clinically relevant medicines appears to be worth pursuing [19].

Regardless of the initial aetiology, ARDS is characterised histologically by diffuse alveolar damage with interstitial and alveolar infiltration of neutrophils and macrophages [20]. The scientific community confirms that thrombolytic activity on fibrin depots in the lungs could enhance the ventilation–perfusion ratio of ARDS and C-ARDS patients [21]. However, these treatments are often administered parenterally, which involves a significant risk of systemic bleeding [22], whereas the inhalation pathway may reduce systemic exposure to fibrinolysis and promote a pulmonary loco-regional effect. The endogenous fibrinolytic system includes plasminogen (PLG) as a key component. PLG is a single-chain plasma protein (MW 92 kDa); it is the zymogen (inactive form) of the serine protease plasmin (fibrinolytic enzyme). PLG activators (t-PA and u-PA) convert PLG to plasmin, which lyses fibrin clots into eliminable products. Additionally, PLG contributes to the regulation of physiological processes like wound healing and operates as an immunomodulator in inflammation [23].

This work is part of a project aiming at consolidating the scientific evidence that supports the clinical pulmonary administration of PLG in ARDS patient. In particular, the work involves the off-label use of a PLG-based eye drop, namely, a human plasminogen orphan medicine (PLG-OMP, Orphan Medicinal Product number EU/3/07/461). PLG-OMP aerosolisation has been preliminary assessed, matching the Ph. Eur. requirements for a solution for aerosolisation [1]. However, part of the enzymatic activity was lost during either ultrasonic or jet nebulisation; it was found a limited correspondence between suitable lung-targeted aerodynamic distribution and activity performance. Since nebulisation techniques can affect the physical–chemical properties of labile drugs by generating heat and shear stresses [24] , the hypothesis of the present work is that mesh nebulisation could provide lower damaging stress but maintain lung-targeted aerodynamic features. Additionally, no previous pulmonary biopharmaceutical assessment of the zimogen protein PLG has been reported in the literature. To form the basis for PLG-OMP clinical off-label administration, a pulmonary biopharmaceutical evaluation is proposed here. Beyond the enzymatic activity, we also hypothesise that it is possible to take advantage of the immunomodulating effect of PLG. To verify the latter hypothesis, the nebulised PLG was tested for the first time on a cellular model of inflammation. The model was developed ad hoc to simulate the administration of the drug on a pre-established inflamed condition.

## 2. Materials and Methods

The workflow of the performed studies is reported in Scheme S1 (Supporting Info File).

### 2.1. Materials

Human plasminogen eye drops 1 mg/mL (PLG-OMP, Orphan Medicinal Product number EU/3/07/461 in EU and Orphan Designation number 10-3092 in US), Kedrion S.p.A. (Gallicano, (Lu) Italy). BIOPHEN™ Plasminogen LRT Ref 22151 (HYPHEN BioMed, Neuville-sur-Oise, France), Human PLG (Plasminogen) ELISA Kit, bicinchoninic acid assay (BCA), Toluene, Acetone, Citric Acid, Diethylenetriaminepentaacetic acid (DTPA), Egg Yolk Emulsion, gelatine from bovine skin type B, RPMI amino acid solution and type II mucin from porcine stomach were purchased from Sigma Aldrich (Milano, Italy). Clioxycarb O_2_/CO_2_ gas 95/5 by mol mixture was purchased from SOL S.p.A. (Monza, Italy). Human lung adenocarcinoma NCI-H441 epithelial cell line was purchased from the American Type Culture Collection LGC standards ((ATCC HTB-174), Milan, Italy) and propagated as indicated by the supplier. The murine macrophage cell line RAW 264.7 was purchased from the Cell Lines Service (Eppelheim, Germany) and propagated as indicated by the supplier. Dulbecco’s phosphate-buffered saline (DPBS), fetal bovine serum (FBS), antibiotics (penicillin/streptomycin), RPMI-1640 medium and Dulbecco’s Modified Eagles Medium (DMEM) were purchased from Sigma Aldrich (Milan, Italy). Cell proliferation reagent (WST-1) was provided by Roche diagnostic (Milan, Italy). Insulin-Transferrin-Selenium (ITS), Dexamethasone, Trypsin-EDTA and Triton X-100 were purchased from Sigma Aldrich (Italy). Haematoxylin and Eosin were purchased from Fluka (Buchs, Switzerland). Normal goat serum (NGS), primary antibody for *Zonula Occludens-1* (ZO-1), primary antibody for Occludin, primary antibody for E-cadherin, Alexa Fluor 594 anti-rabbit and Alexa Fluor 488 anti-mouse were purchased from Thermo Fisher Scientific (Waltham, MA, USA). Hank’s buffer salts solution (HBSS10X) was obtained from Corning, Manassas (VI, USA) and 1 to ten diluted with deionised water when necessary. Multiplex LEGENDplex Mouse Macrophage/Microglia Panel Detection was obtained from Biolegend (Campoverde, Italy). ProlongTM Diamond Antifade Mountant with DAPI were purchased from Thermo Fisher Scientific (Waltham, MA, USA).

### 2.2. Plasminogen (PLG-OMP) Aerosolisation

Aliquots of 5 mL of PLG-OMP were atomised using the Aerogen Solo^®^ mesh nebuliser (Galway, Ireland). Nebulisation was performed in either air or oxygen-rich conditions by using 10 L/min flow of compressed air or 95% O_2_ and 5% CO_2_ molar mixture (clioxycarb), respectively. Nebulisation was conducted until no cloud was produced. Atomised samples were collected by condensation into a glass bioaerosol impinger (0–4 °C) [25]. Both the residual solutions in the nebuliser cup and the collected nebulised aliquots were submitted to biochemical characterisation (enzymatic activity and electrophoretic SDS-PAGE) and compared to the untreated PLG-OMP solutions (CTRL).

The presence of protein aggregates or fragments of PLG was evaluated by dynamic light scattering (DLS—Zetasizer Nano ZS, Malvern Panalytical Ltd., Malvern, UK). Protein analysis was performed by setting proteins as the material (RI 1.450; Absorption 0.001) and water as the dispersant (T 25 °C; RI 1.330), with a display range of 0.1–6000 and a threshold of 0.05–0.01. The molecular weight of the main protein peak and its mass percentage were obtained based on scattering intensity data. Protocol validation is reported in the Supplementary Information File (Figure S1).

### 2.3. Maintenance of Enzymatic Activity

A Plasminogen chromogenic kit (BIOPHEN™ Plasminogen LRT, HYPHEN BioMed) was applied to evaluate the PLG activity maintenance after nebulisation. The assay involves two reagents: streptokinase (R1) and a chromogenic substrate (R2). The PLG activity is measured following its specific activation by the addition of streptokinase; the complex plasminogen-streptokinase cleaves the plasmin-specific substrate SPm41, releasing para-nitroaniline (pNA) and absorbing at 405 nm. PLG activity was calculated on a calibration curve built by using untreated PLG-OMP 10–80 µg/mL samples (R^2^ = 0.9902). A bicinchoninic acid assay (BCA) was used for the quantification of the protein content (PLG-OMP calibration curve 5–80 µg/mL; R^2^ = 0.9997). The maintenance of the enzymatic activity of the nebulised samples was expressed as reported below, where the reference PLG-OMP product corresponded to 100% activity.

(%) = 100 × (µg/mL of active protein (Activity Assay))/(µg/mL of total protein (BCA)) (1)

### 2.4. Protein Characterisation

SDS-PAGE was used to determine the native glutamic acid-plasminogen (Glu-PLG) and lysine-plasminogen (Lys-PLG). SDS-PAGE was performed under denaturing conditions with a 4–12% gel (Invitrogen, Carlsbad, CA, USA). PLG samples and home-made PLG standard were prepared with an LDS sample buffer 4× (Invitrogen) denaturing agent, followed by 5 min of heat denaturation at 95 °C. The gel was loaded with 4 µg of protein PLG samples, and the electrophoretic run lasted 55 min at 200 volts. The gel was captured using Chemidoc MP by the ImageLab software Lys-PLG. The results were expressed as a percentage of the band assigned to Glu-PLG or Lys-PLG in comparison to all bands in the line.

### 2.5. Droplet Distribution of Nebulised PLG-OMP

The aerodynamic evaluation of the aerosol clouds was performed by using a glass twin stage impinger (TSI), according to European Pharmacopoeia X ed., 2.9.18 Apparatus A procedure [26]. The B portion of the impinger was connected to the T-piece junction of the mesh nebuliser and a mechanical pump (Edwards RV5, Irvine, CA, USA) provided 60 L/min air flow. Fine droplets were captured between the TSI and the pump on a propylene filter (low resistance filter, PARI) and a cold trap. The upper and lower chambers of TSI were filled with 7 mL and 30 mL of 0.2 µm filtered Milli-Q water, respectively. PLG-OMP (5 mL) was placed into the nebuliser cup. Before each nebulisation, 60 ± 5 L/min flow were guaranteed at the inlet to the throat, as measured with a flow meter (Brooks Instrument, Hatfield, PA, USA). The pump was started and, after 10 s, the nebuliser as well. At the end of nebulisation, the upper and lower stage solutions were collected individually and then washed with Milli-Q water. The collected protein was quantified by BCA. The procedure was repeated six times. The results for each of the two parts of the apparatus were expressed as a percentage of the total amount of active substance.

### 2.6. Penetration of Aerosolised PLG through Artificial Mucus

An in vitro mucus model was set, as reported by Costabile et al. 2016 [27]. The first to propose this composition was Ghani et al. [28], who developed a simulated mucus based on patients’ sputum. Briefly, 1 mL of gelatine solution (10% *w*/*v*) was placed in each well of a 24-well plate, hardened at room temperature and stored at 4 °C until use. The artificial airway mucus was prepared by adding 250 µL of sterile egg yolk emulsion, 250 mg of mucin, 0.295 mg DTPA, 250 mg sodium chloride, 110 mg potassium chloride and 1 mL of RPMI to 50 mL of water [29]. Then, 1 mL of artificial mucus was placed on each of the hardened gelatine gels. PLG-OMP was directly nebulised for 30 s on each artificial mucus sample, and the plates were incubated maintaining 100% relative humidity so as to simulate a wet environment. At regular time intervals, the artificial mucus was withdrawn and separated from the gelatine gel. The PLG content in the gelatine and artificial mucus was quantified by specific Human PLG ELISA assay (Sigma-Aldrich).

### 2.7. Biological Investigation

In vitro biological evaluations were conducted on lung epithelial NCI-H441 cell line and on macrophage RAW 264.7 cell line. NCI-H441 cells were grown in RPMI-1640 medium (RPMI) with 1% pen/strep and 10% FBS at 37 °C in a 5% CO_2_ atmosphere, while RAW 264.7 cells were grown in Dulbecco’s Modified Eagles Medium (DMEM) with 1% pen/strep and 10% FBS at 37 °C in a 5% CO_2_ atmosphere.

#### 2.7.1. PLG Permeability across an In Vitro Model of Pulmonary Epithelium

##### Cytotoxicity Screening on NCI-H441 Cells by Tetrazolium Salts Assay (WST-1)

Cell viability studies were carried out on the NCI-H441 cell line in 96-well plates using the WST-1 test [30]. The cells were seeded in 96-well plates (4 × 10^4^ cells/well). After 24 h, the culture medium was removed and replaced with test samples (PLG-OMP in the range of 4–900 μg/mL). After 4 h of incubation, media were removed, cells were washed twice with PBS and tetrazolium salt WST-1 (1/10 in medium) was added. The produced formazan was quantified after 4 h incubation at 450 nm with a microplate reader (BioTek 800/TS, Thermo Scientific, Walthman, MA, USA). Finally, the half-maximal inhibitory concentration (IC50) was determined.

##### In Vitro Air–Liquid Interface (ALI) Model

NCI-H441 cells were seeded onto a Transwell^®^ insert (2.5 × 10^4^ cells/well), and 600 µL of full medium was added into the basolateral chamber. After 48 h, the apical medium was removed (100 µL), leaving the apical surface of the cells exposed to air (air–liquid culture) while that of the basolateral chamber was replaced with cell medium containing 1% insulin transferrin selenium (ITS) and 200 nM dexamethasone. Cell monolayer formation was monitored by measuring the transepithelial electrical resistance (TEER) using a voltmeter with rod electrodes (Voltmeter Millicelles-ERS, Millipore, Molsheim, France). Monolayer formation and differentiation were verified by microscopy analysis of haematoxylin/eosin-stained samples and immunofluorescence analyses. For haematoxylin/eosin staining, the monolayers were washed with deionised water for 5 min, then haematoxylin (0.7% *w*/*v*, sodium iodate, aluminium ammonium sulphate 12 H_2_O) was added and the mixture was incubated for 5 min. After washing with water, the samples were treated for 2 min with eosin (0.5% *w*/*v* in acidified 90% ethanol) for cytoplasm staining. Immunofluorescence staining of adhesion molecules, i.e., Zonula occludens-1 (ZO-1), E-cadherin and Occludin, was performed [31]. NCI-H441 monolayers were first fixed with 3.8% paraformaldehyde in PBS 1× for 1 h, treated with PBS 1×//Triton X-100 solution (0.2% *v*/*v*) for 15 min and blocked with normal goat serum (NGS, 1% *w*/*v* in PBS 1×) for 30 min. Anti-E-cadherin (1:100 in NGS), anti- ZO-1 (1:500) and anti-Occludin (1:50 in NGS) were applied separately on individual monolayer samples and incubated overnight at 4 °C. After washing with NGS solution (1% *w*/*v* in PBS 1×), samples were incubated for 2 h in the dark with the secondary antibody. Finally, samples were mounted on slides (ProlongTM Diamond Antifade Mountant with DAPI, Thermo Fisher Scientific). The samples were observed with a fluorescence laser scanning microscope (Nikon Eclipse Ts2R, Tokyo, Japan) using the following wavelengths: E-cadherin and Occludin λ_ex_ 499 nm/λ_em_ 519 nm, λ_ex_ 591 nm/λ_em_ 618 nm for ZO-1 and λ_ex_ 345 nm/λ_em_ 455 nm for nuclei (DAPI).

##### Assessment of Nebulised PLG Permeation through ALI Monolayer

**Figure 1:**
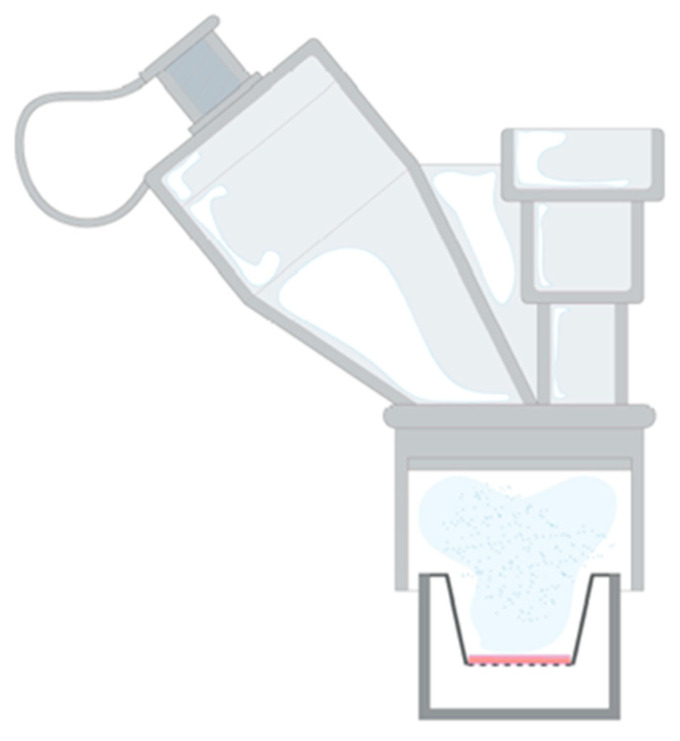
Setup for the direct nebulisation on an ALI monolayer with the Aerogen^®^ Solo^TM^ mesh nebuliser.

Firstly, a cytotoxicity evaluation was performed. PLG-OMP was nebulised for 30 s directly on the apical portion of the NCI-H441 monolayers (*n* = 3) (Figure 1). The deposited PLG corresponded to 30.0 ± 0.06 µg of protein. Nebulised samples were then incubated for 2 h with 600 µL of HBSS 1×, placed in the basolateral chamber. At the end of the incubation time, the monolayers were rinsed with PBS 1×, and tetrazolium salt WST-1 (1/10 in medium) was added. The formazan produced was quantified at 450 nm.

For permeation experiments, PLG-OMP was nebulised for 30 s on NCI-H441 ALI monolayers (*n* = 16) and then incubated with 600 µL of HBSS 1× in a basolateral chamber. At regular time intervals (30, 60, 90, 120 min), the HBSS was withdrawn and the permeated plasminogen was quantified by PLG ELISA assay (Sigma-Aldrich). Apparent permeability (Papp) was calculated from the linear correlation observed on a cumulative concentrations vs. time plot:

Papp = ((dC/dt)V)/(AC_0_), (2)

where dC/dt is the line slope, V is chamber volume, A is the monolayer area and C_0_ is the PLG concentration in PLG-OMP. The experiment was performed under sink conditions.

#### 2.7.2. Immunomodulating Activity of Nebulised PLG

At first, cell viability studies were carried out on the RAW 264.7 cell line. Cells were seeded in 24-well plates at 10^6^, 6.5 × 10^5^ and 5 × 10^5^ cells/well for cytotoxicity studies at 6, 18 and 24 h respectively. Then, 24 h after seeding, the culture medium was removed and replaced with test samples, either 5–20–50 μg/mL of PLG or 1 μg/mL LPS. After incubation, media were removed, and tetrazolium salt WST-1 (1/10 in medium) was added after washing with PBS 1×. The formazan produced was quantified at 450 nm with a microplate reader. Preliminary RAW 264.7 activation assays were performed using LPS in the range of 0.2–1 μg/mL. Cells (5 × 10^5^) were seeded in 24-well plates and left to proliferate for 24 h prior to the incubation with LPS samples, which lasted a further 24 h. At the end of the incubation time, the supernatant was collected and analysed using the LEGENDplex Mouse Macrophage/Microglia Panel Detection multiplex system (Campoverde, Italy) for TNF-α, IL-1β and IL-6 quantification.

The evaluation of the maintenance of PLG immunomodulatory activity was performed as follows. RAW 264.7 cells were seeded in 24-well plates (10^6^ cells/well) and left to proliferate for 24 h at 37 °C, 5% CO_2_. The cells were incubated with LPS (0.2 μg/mL). After 6 h, PLG (5 μg/mL) and the nebulised PLG collected on a glass bioaerosol impinger (5 μg/mL) were added and incubated for additional 18 h. Cells treated with medium and with LPS only (0.2 μg/mL) were used as the negative and positive controls, respectively. At the end of the incubation time, the supernatant of each sample was collected and centrifuged at 300× *g* for 10 min at 4 °C. The obtained supernatants were evaluated for TNF-α, IL-1β and IL-6 quantification (LEGENDplex Mouse Macrophage/Microglia Panel, Campoverde, Italy). Data were normalised according to the total protein content. Cells were lysed with 100 µL of Ripa buffer, kept for 30 min at 4 °C under agitation and then centrifuged at 1000× *g* for 10 min. The supernatant was collected, and the total protein content was determined by BCA assay. Bovine serum albumin (BSA) was used as the standard for the calibration curve (2–100 μg/mL; R^2^ = 0.9994).

### 2.8. Statistical Analysis

All the experiments were performed at least six times unless otherwise specified. Data are means ± SEM. The statistical significance between groups was determined by one-way ANOVA for paired samples, followed by Tukey–Kramer post hoc test. A *p* < 0.05, 0.001 < *p* < 0.01 and *p* < 0.001 were considered significant, very significant and extremely significant, respectively.

## 3. Results

### 3.1. PLG Aerosolisation under Air or Oxidising Condition

The nebulisation of 5 mL of PLG-OMP was nearly complete, with only a small amount of lather on the device walls, corresponding to 0.146 ± 0.003 mg of undelivered protein. The aerosolisation took 26.8 ± 6.3 min at a flow rate of 0.19 ± 0.04 mL/min, in agreement with the user manual of Aerogen^®^ Solo^TM^. Furthermore, the collected nebulised samples, obtained under both air and oxygen conditions, showed no protein aggregates, with more than 98% of PLG remaining in its monomeric form (Figure S2, Supplementary Information File) and a calculated MW of 99.6 ± 3.7 KDa (nebulised in airflow) and 99.6 ± 5.2 KDa (nebulised in oxygen flow), in agreement with PLG-OMP data (MW 93.9 ± 4.2 kDa) tabulated MW (92 KDa) [32]. Concerning the enzymatic activity, it was unaffected by mesh nebulisation in air, with a percentage of activity maintenance of 93.8% ± 11.5 with respect to untreated PLG-OMP. In contrast, the presence of oxygen flow affected the activity of the protein, resulting in 59.4% ± 15.9 of activity maintenance with respect to untreated PLG-OMP.

In the aerosolised clouds, the distribution of the two isoforms, Glu-PLG and Lys-PLG, was assessed by SDS-PAGE. PLG-OMP is mainly composed of the natural glycoforms of plasminogen, Glu I and Glu II, having glutamic acid as the first amino acid in the chain (Glu-PLG). Lys-PLG is the most common shortened version of PLG, missing the first 77 amino acids and having lysine as the first amino acid of the chain. Lys-PLG is regarded as a product impurity or an undesirable pre-activation form. The Glu-PLG migrates as two distinct bands corresponding to the two glycoforms: Glu I and Glu II; if present, the Lys-PLG is seen as two separate distinct bands, Lys I and Lys II, derived from Glu I and Glu II, respectively. The replicates were run on gel and compared to standards (Figure S3 in the Supplementary Information File). The percentage of protein bands related to Lys-Plasminogen was found to be 0% in all examined samples, whereas the bands of Glu-PLG forms were not altered for all samples (Table 1).

An aerodynamic assessment of the fine particles in the PLG-OMP aerosol cloud was performed using a glass impinger apparatus [26] and by dosing the PLG impacting in each TSI component by BCA assay. Most of the nebulised protein, corresponding to 90.5% ± 14.6 of the total delivered dose, impacted the lower impinger stage. Beyond the ordinary aerodynamic particle cut-off size of 6.4 μm between the two stages [33], the TSI components can be divided into sub-regions correlating to specific tracts of the respiratory tree, such as oral cavity, pharynx and larynx of the upper respiratory tract, bronchi and the entire targeted lung area (lower respiratory tract) [34]. By adopting such a distribution, the delivered PLG could be portioned into percentages of protein impacting specific respiratory portions (Table 2). As expected, the highest amount of protein was found in the portion corresponding to lung.

### 3.2. Aerosolised PLG Mobility in Simulated Mucus

**Figure 2:**
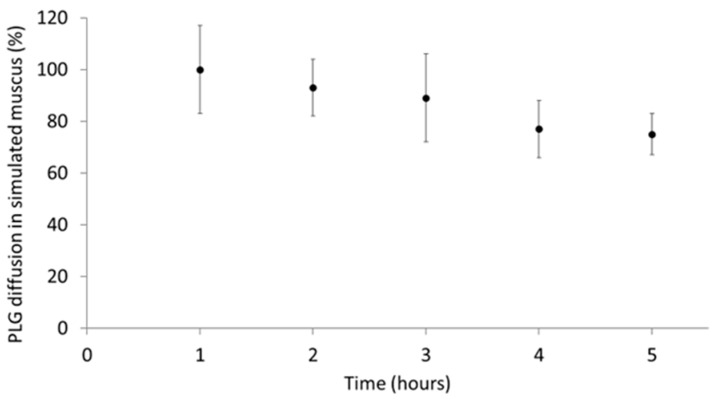
Diffusion kinetics of nebulised PLG in artificial mucus on a gelatine layer. PLG-OMP was deposited on the air side of the mucus layer, and every hour, both mucus and gelatine were treated to quantify the PLG content (ELISA). The diffusion percentage corresponds to the amount of PLG reaching the gel layer underneath.

To evaluate the tendency of PLG to diffuse through airway mucus, PLG-OMP was directly nebulised on an in vitro model consisting of a layer of artificial mucus deposited on solid gelatine [35]. As reported in Figure 2, PLG progressively diffused from the upper mucus layer to the lower gelatine gel, reaching a cumulative 20% value after 5 h (*p* < 0.05). The plasminogen was then distributed homogenously in the artificial mucus layer, passing for 1/5 in the gel layer below.

### 3.3. PLG Permeability across a Monolayer

**Figure 3:**
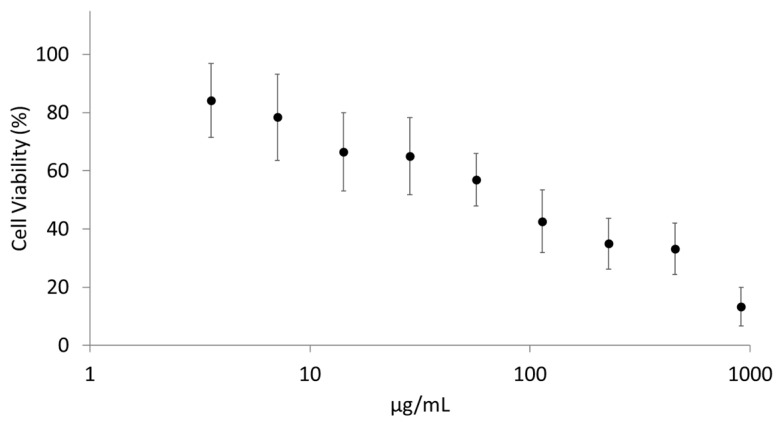
Cytotoxicity screening performed on NCI-H441 cells, exposed to PLG-OMP in the 4–900 μg/mL PLG concentration range. The values indicated in the figure are means ± SD (*n* = 8).

Firstly, cytotoxicity screening was performed on the NCI-H441 cell line. Exposing it to different PLG-OMP concentrations (4–900 µg/mL) showed an inhibiting concentration (IC_50_) corresponding to 58.4 µg/mL (Figure 3). Untreated cells were used as the control.

NCI-H441 cells are frequently used in drug disposition studies due to their ability to form confluent monolayers of polarised cells with high TEER [36]. The NCI-H441 monolayer was obtained in 8 days, as demonstrated by the TEER values. TEER was monitored from day 4 to day 12. An increase of the values was noticed, which then stabilised at around 600 Ω on the 8th day; the values then remained stable until the 11th day, and finally decreased on the 12th day (Figure S4a). Staining with haematoxylin/eosin was performed in order to evaluate the confluence of the cell monolayer. It was seen that at 7 days of culture, the cells were not yet able to form a complete monolayer (Figure S4b in the Supplementary Information File), while at 8 days of culture, the cells completely covered the surface of the Transwell^®^ filter (Figure S4c). Furthermore, with prolonged culture times (10 and 12 days), the formation of a cell multilayer was observed (Figure S4d,e in the Supplementary Information File). In order to confirm cell differentiation by the presence of tight junction expressions, immunofluorescence was performed at 8 days of culture on NCI-H441 cultured in ALI conditions. A specific perimeter localisation of ZO-1, E-cadherin and Occludin expression, associated with cell differentiation, was observed (Figure S5).

PLG-OMP was directly nebulised on the ALI monolayer as a preliminary evaluation. The monolayer was placed in the second chamber of the TSI to collect the inhalable PLG droplets. However, the nebulisation time required to impact a measurable amount of PLG-OMP was too long, compromising the stability of the monolayer (data not displayed). As such, the direct PLG-OMP nebulisation on the Transwell^®^ (Figure 1) was preferred. The biocompatibility of PLG-OMP directly nebulised on the ALI monolayer for 30 s was preliminarily verified (Figure S6 in the Supplementary Information File). The maintenance of TEER values over 600 Ω confirmed the integrity of the monolayer after PLG-OMP nebulisation and throughout the entire permeation experiment.

**Figure 4:**
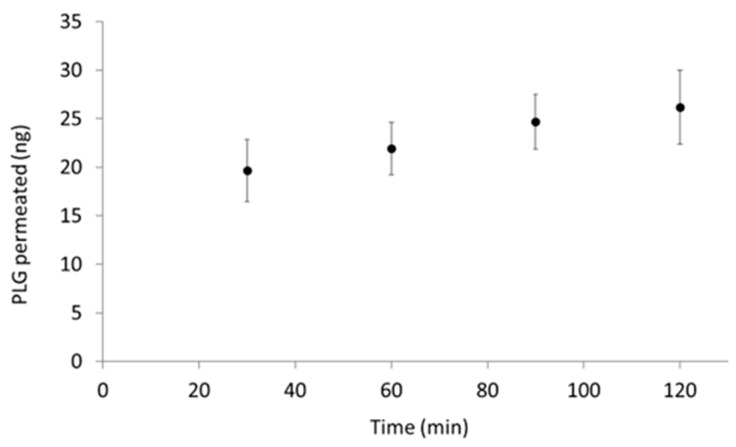
Permeation kinetics of nebulised PLG through the NCI-H441 ALI monolayer over time. PLG-OMP was directly nebulised on an in vitro model of the human alveolar epithelium [31]. At regular time intervals (30, 60, 90 and 120 min), the HBSS medium from the basolateral chamber was withdraw, and the PLG concentration was then determined by specific ELISA assay.

The permeation assay was carried out by nebulising PLG-OMP for 30 s (corresponding to 30.0 ± 0.06 µg of deposited protein) on each monolayer. PLG permeation was monitored using a specific Human PLG ELISA assay. A slow but progressive increase of PLG concentration was observed in the basolateral chamber, reaching a cumulative value of 26 ng after two hours, corresponding to 0.08% of the total deposited protein (Figure 4). The apparent permeability (Papp) of PLG across the monolayer was calculated as 3.82 × 10^−9^ cm/s.

### 3.4. Maintenance of PLG Immunomodulatory Activity

A preliminary cell viability study performed on the RAW 264.7 cell line showed the absence of cytotoxicity for each PLG tested concentration, as well as for LPS 1 μg/mL (Figure S7 in the Supplementary Information File). Firstly, the LPS concentration screening (0.2–1 μg/mL) evidenced that the minimum concentration capable of stimulating the production of cytokines during 24 h incubation was LPS 0.2 μg/mL (Figure S8 in the Supplementary Information File). It was noticed that the TNF-α concentration values were not affected by increasing LPS concentration, resulting in a plateau. Differently, a dose–response relationship was evident for IL-6. An LPS concentration of 0.2 μg/mL, i.e., the lowest tested concentration capable of stimulating the macrophage cell line, was thus adopted for further studies.

**Figure 5:**
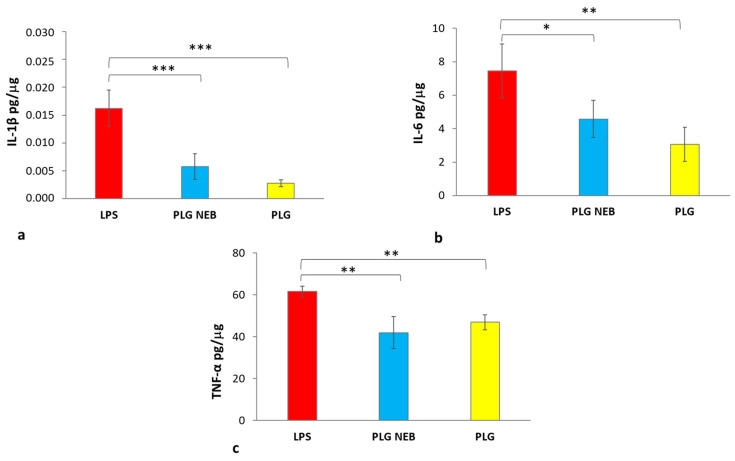
Maintenance of an immunomodulating effect on RAW 264.7 cells of nebulised PLG-OMP: assessment of pro-inflammatory cytokine secretion induced by 24 h stimulation with LPS (red), stimulated with LPS for 24 h and treated with either nebulised PLG-OMP (blue) or PLG-OMP (yellow). PLG NEB: aerosol cloud collected in a bio-impinger. PLG: untreated eye drops. (**a**) Expression of IL-1β, *** *p* < 0.001 vs. PLG NEB and PLG; (**b**) expression of IL-6, * *p* < 0.05 vs. PLG NEB, ** 0.001 < *p* < 0.01 vs. PLG; (**c**) expression of TNF-α, ** 0.001 < *p* < 0.01 vs. PLG NEB and PLG. Data were normalised in relation to the total protein concentration and expressed as pg of cytokine per μg of protein.

RAW 264.7 cells were treated with LPS (24 h) and the nebulised PLG, collected on a glass bioaerosol impinger, was added 6 h post LPS addition. LPS alone and LPS with PLG-OMP eye drops (not nebulised) were used as a reference. From the results (Figure 5), it was observed that both PLG-OMP and the nebulised PLG-OMP reversed the effects of LPS activation by reducing the expression of pro-inflammatory cytokines IL-1β, IL-6 and TNFα. Notably, for IL-1β, both the nebulised product and PLG-OMP treatments significantly reduced its release (IL-1β 0.006 pg/µg and 0.003 pg/µg, respectively), compared to the positive control (IL-1β value of 0.016 pg/µg) (Figure 5a). Moreover, it was found that the nebulised product and PLG significantly decreased IL-6 secretion compared to the positive control (IL-6 values of 4.6 pg/µg and 3.07 pg/µg, respectively as compared to 7.6 pg/µg) (Figure 5b). The release of TNF-α from activated macrophages treated with either the nebulised product or PLG-OMP was also significantly affected, resulting in 41.9 pg/µg and 46.9 pg/µg, respectively. Those values were lower than what was recorded for the positive control (61.7 pg/µg) (Figure 5c).

## 4. Discussion

Pulmonary delivery of PLG, through the off-label administration of PLG-OMP eye drops, has been considered as a possible therapy for C-ARDS patients [1]. During the COVID-19 pandemic, several drugs acting on the coagulopathy aspect of the disease were investigated. Mostly, heparin or tissue plasminogen activator/streptokinase were adopted under conventional routes of administration (sc. or i.v.), but a few researchers also considered the inhalation route. Due to pandemic resolution, study NCT04842292 [37], on nebulised heparin for COVID-19-associated Acute Respiratory Failure, terminated due to a lack of enrolment, whereas INHALE-HEP (NCT04723563) [38] was completed, although no results have been made available yet. Previous studies of patients with serious breathing problems due to pneumonia and other conditions found that nebulised heparin reduced the formation of small blood clots in the lungs, reduced the amount of injury to the lungs and hastened recovery, allowing patients to return home more quickly [39,40]. In 2019, a randomised controlled trial on nebulised streptokinase versus unfractionated heparin was conducted on patients with ARDS [41]. The study concluded that inhaled streptokinase could serve as rescue therapy in patients with severe ARDS. The therapy was found to improve oxygenation and lung mechanics more quickly than heparin or conventional management.

From a pharmaceutical technology perspective, nebulising a protein solution requires the monitoring of protein stability, the determination of droplet distribution and verification of activity maintenance. The generation of an aerosol exposes solutions and their components to physical stresses, often inducing protein unfolding and thermal degradation [42]. It is surprising that despite the presence of literature evidence of the effective clinical administration of nebulised proteins, reported descriptions of the used nebulisation devices have been poor. More efforts to indicate the most suitable administration setting in terms of the nebulisation device would improve the bench to bed translation of new medicines and off-label applications of already marketed ones. In a previous work, the jet and ultrasonic aerosolisation of PLG-OMP resulted in a significant loss of enzymatic activity. Only when the jet aerosolisation was set to the lowest air pressure, it was possible to preserve 89.8% of PLG proteolytic activity. However, the median diameter value (dv_(50)_) resulted 9.2 ± 1.0 µm, which is not suited for pulmonary delivery [1]. Vibrating-mesh nebulisers, like the Aerogen^®^ Solo^TM^, employ perforated plates that vibrate to generate aerosols. Several studies have shown that no significant heat develops during atomisation, and the provided shear stress is lower than that of jet nebulisers. These devices are generally useful for delivering labile molecules, such as proteins [43]. In the present study, the mesh nebulisation of PLG-OMP was effective in preserving the chemical and physical features of PLG. Analyses of the protein in the collected clouds evidenced the maintenance of PLG in its monomeric form, with production of neither aggregates nor fragments, as demonstrated by DLS evaluations (Figure S2 in the Supplementary Information File). Moreover, the Glu-PLG glycoforms were maintained, with no cleavage to Lys-PLG (Table 1). The absence of Lys-PLG in aerosolised samples confirms the stability of PLG following mesh nebulisation. Regarding protein activity maintenance, it was totally preserved under normal aerosolisation conditions. Assuming that in cases of critical patient conditions, the off-label administration of PLG-OMP could occur in the presence of oxygen supply, the aerosolisation of PLG-OMP was also performed under an oxygen gas flow [44]. The nebulisation of PLG-OMP in the presence of oxygen led to significant decrease of PLG enzymatic activity (activity maintenance of 59.4% was recorded). Proteins are highly sensitive to oxidation, notably due to the modification of sulphur-containing amino acids Cys and Met, aromatic amino acids Trp, Tyr and Phe, as well as His, Pro, Lys and Arg [45]. The effect of oxidation depends on the oxidising stimuli and on the protein structure, varying from an effect on protein folding to the fragmentation of the sequence [46]. According to our DLS analysis, the protein was neither significantly aggregated nor fragmented, and the SDS-PAGE investigation evidenced no change in the PLG isoform distribution. Thus, the loss of activity may be attributed to the oxidative impact of oxygen on amino acid residues [47] essential for the enzymatic activity of PLG; however, the sequencing of the protein to define which amino acids are involved is beyond the scope of the present paper. 

Aerosolised PLG showed an optimal in vitro deposition profile, with approximately 90% of the active ingredient impacting in the lower portions of the impinger. Under the Ph. Eur. setting, i.e., using a flow rate of 60 L/min through the impinger, the particle cut-off between upper and lower impingement chambers was 6.4 μm, indicating that particles smaller than 6.4 μm passed into the lower compartment [33]. The collected data are in agreement with previous reports on Aerogen^®^ Solo^TM^ mesh nebulisation, which describe mass median aerodynamic diameters of 4.4 μm and 2.7 μm for Aerogen^®^ Solo^TM^ and Aerogen^®^ Solo^TM^ with a T-piece device, respectively [48,49,50]. The results confirm that the clouds provided by these devices mainly impact the lower TSI compartment, suggesting the suitability of this approach for lung delivery in in vivo administration [34].

Two main aims of the future pulmonary administration of PLG are to take advantage of its loco-regional fibrinolytic action and to limit adverse effects due to systemic administration. To this end, airway mucus should not represent a barrier to PLG mobility, and PLG systemic absorption through alveolar permeation should be limited.

The mobility of nebulised drugs in artificial mucus is usually tested to verify that the layer of mucus that covers the respiratory epithelium does not represent a real physical barrier. The observed time-dependent transport of PLG through the simulated mucus suggested its homogeneous distribution toward the bronchoalveolar epithelium. The mobility observed for nebulised PLG was in agreement with literature reports, falling in between nanosized carriers, displaying about 5% penetration after 6 h [35], and small molecule drugs, reaching about 70% after 5 h [27].

The immortalised human lung adenocarcinoma cell line NCI-H441 (ATCC HTB-174), comprising epithelial lung distal cells, was selected in this study for biological evaluations [51]. The NCI-H441 cell line is frequently used in drug disposition studies due its ability to form confluent monolayers of polarised cells with high TEER [36], making it a valid in vitro model for permeation studies [31,51]. The permeability obtained for PLG (Papp 3.82 × 10^−9^ cm/s) is coherent with the Papp values determined for macromolecules with analogous molecular weights [36]. Low permeability of inhaled drugs is needed to extend their residence time in the lung, subsequently diminishing their diffusion through the lung epithelium toward the systemic circulation [52]. Thus, the low Papp value and the low amounts of PLG recovered in the basolateral chamber suggest a limited systemic absorption in vivo, supporting a hypothesised loco-regional enzymatic action.

Due to its main role as a zymogen of plasmin, PLG is closely associated with fibrinolysis, but PLG, its activators and its receptors play roles in various inflammation regulatory processes [53]. From this viewpoint, PLG administration could also help in the resolution of the severe lung inflammation that is typical of ARDS by direct interaction with macrophages. Macrophages have the ability to change into different phenotypes during inflammation, with a timely M1–M2 progression accompanying the inflammatory phases and resolution. M1 macrophages are involved in the beginning of inflammation and are responsible for inflammatory signalling, while M2 macrophages produce anti-inflammatory cytokines, thereby contributing to tissue healing [54]. Vago et al. [55] reported that the PLG receptors that are exposed on the macrophage cell surface bind plasminogen to enhance plasminogen activation. PLG and PLG receptors regulate key steps in the resolution of inflammation by affecting monocyte/macrophage migration, reprogramming macrophages toward an M2-like phenotype and reducing pro-inflammatory cytokine release.

LPS stimulation of RAW 264.7 macrophage cell line is generally applied as an in vitro model of inflammation [54,56], and LPS stimuli is also applied for in vivo ARDS animal models [57]. To simulate the in vivo effect of PLG treatment in an already induced inflammatory state, RAW 264.7 cells were treated with LPS for 24 h, but PLG was applied 6 h post LPS addition. Under the set conditions, PLG and LPS were simultaneously present for 18 h. It is known that LPS increases the production of urokinase-type plasminogen activator (uPA) in RAW 264.7 cells through the uPA core promoter domain [58]. uPA converts PLG into plasmin, and the plasmin produced can activate plasminogen/plasmin and plasmin/protease-activated receptor (PAR)-1 to modulate the inflammatory condition [59]. As reported in Figure 5, nebulised PLG treatment significantly reduced the production of relevant inflammatory mediators such as IL-1β, IL-6 and TNF-α in LPS-stimulated macrophages. Concerning the expression values of the investigated cytokines, striking differences were observed for TNF-α expression compared to those of IL-6 and IL1-β. According to the literature, the stimulus of LPS causes an immediate release of TNF-α, while IL-6 reached a peak after 6 h, mediated by TNF-α rather than LPS directly, indicating a cascading pattern after LPS stimulation in RAW 264.7 macrophages. This phenomenon may support the obtained results, where TNF-α showed elevated concentrations with respect to IL-6 [60]. Notably, the immunomodulating effect of PLG was evident for all tested cytokines and was preserved in the nebulised product. In addition, using human plasminogen minimises the risk of an immune response to non-human proteins, which could cause severe asthma/anaphylactic reactions when inhaled.

## 5. Conclusions

PLG-OMP mesh nebulisation effectively produced inhalable droplets for lung delivery of active PLG. In vitro investigations suggested a good safety profile of inhalable PLG, excluding high systemic absorption but indicating good mucus diffusion. The delivered protein maintained both its enzymatic activity and immunomodulating effect toward macrophages, which were activated by LPS inflammatory stimuli. All physical, biochemical and biopharmaceutical assessments of mesh aerosolised PLG-OMP are evidence for the potential of its off-label administration as treatment for ARDS patients. The locoregional deposition of PLG could provide fibrinolytic and anti-inflammatory actions, contributing to ARDS treatment. In vivo animal assessment represents the next step and will provide additional knowledge on PLG-OMP efficacy and dose correlations. Due to the simplicity of the PLG-OMP formulation (it is an eyedrop solution), the protein results susceptible to oxidation when the administration in oxygen current was simulated . Thus, in view of future clinical application, the concomitant administration of PLG-OMP aerosol and oxygen is discouraged, as it may result in a significant loss of enzymatic activity.

## Figures and Tables

**Table 1:** SDS-PAGE investigation: percentage distribution of glycoforms Glu-I, Glu-II, Lys-I and Lys-II in nebulised PLG-OMP collected in a glass bioaerosol impinger under air (Neb PLG air) and oxygen (Neb PLG ox) currents. The samples (*n* = 4) were compared to untreated PLG-OMP (CTRL) and standards (STD Glu PLG, STD Lys PLG).

Samples	BAND% ± SD%			
	Glu-I	Glu-II	Lys-I	Lys-II
STD Glu PLG	66	35	-	-
STD Lys PLG	-	-	62	38
CTRL	65	35	-	-
Neb PLG air	64 ± 4.5	36 ± 7.9	-	-
Neb PLG ox	59 ± 8.8	39 ± 10.3	-	-

**Table 2:** Nebulisation of 5 mL of PLG eye drops using Aerogen^®^ Solo^TM^ and air flow 10 L/min: percentage distribution of nebulised PLG in TSI portions according to the simulation of the respiratory tract.

Simulation of the Nebulised Protein Distribution	
TSI Portion	% ± SD
Junction (T-piece)	11.5 ± 9.7
Throat	0.2 ± 0.2
Lower respiratory tract	80.8 ± 5.7
Exhaled	10.2 ± 9.2

## Data Availability

The data presented in this study are available on request from the corresponding author.

## Supplementary Materials

The following supporting information can be downloaded at: https://www.mdpi.com/article/10.3390/pharmaceutics15061618/s1. Scheme S1. Workflow of the study. Protocol validation for protein aggregates and fragments [32,61,62], Figure S1. Correlation graph of Debye analysis, Figure S2. Protein Size distribution, Figure S3. SDS-PAGE assay, Figure S4. Micrograph of NCI-H441 cell monolayers, Figure S5. Representative fluorescence microscopy images of NCI-H441 cell ALI monolayers. Figure S6. Monolayer viability, Figure S7. Cell viability of RAW 264.7 cells at 6, 18 and 24 h, exposed to 5–20–50 µg/mL of PLG-OMP and LPS 1 µg/mL, Figure S8. Cytokines production by RAW 264.7 cells stimulated with different concentrations of LPS (0.2, 0.5, 0.8 and 1 µg/mL).
