# Supplementary material for: Biopharmaceutical Assessment of Mesh Aerosolised Plasminogen, a Step towards ARDS Treatment
Source: Pharmaceutics. 2023 May 30;15(6):1618. doi: 10.3390/pharmaceutics15061618 (PMC10300680; doi:10.3390/pharmaceutics15061618)
Supplement: Supplementary file 1 [file pharmaceutics-15-01618-s001.zip › pharmaceutics-2280378-supplementary.pdf]

Supplementary information file

Biopharmaceutical Assessment of Mesh Aerosolised Plasminogen, a Step towards ARDS Treatment

Lucia Vizzoni [1,2,†], Chiara Migone [1,†], Brunella Grassiri [1], Ylenia Zambito [1,3], Baldassare Ferro [4], Paolo Roncucci [4], Filippo Mori [5], Alfonso Salvatore [5], Ester Ascione [5], Roberto Crea [5], Semih Esin [6,7], Giovanna Batoni [6,7] and Anna Maria Piras [1,7,*]

[1] Department of Pharmacy, University of Pisa, 56126 Pisa, Italy
[2] Department of Life Sciences, University of Siena, 53100 Siena, Italy
[3] Research Centre for Nutraceutical and Healthy Foods "NUTRAFOOD", University of Pisa, 56124 Pisa, Italy
[4] Anestesia e Rianimazione, Azienda USL Toscana Nord Ovest, 57124 Livorno, Italy
[5] Kedrion S.p.A., Via di Fondovalle, Loc. Bolognana, 55027 Gallicano, Italy
[6] Department of Translational Research and New Technologies in Medicine and Surgery, University of Pisa, 56126 Pisa, Italy
[7] Centre for Instrument Sharing of University of Pisa (CISUP), 56126 Pisa, Italy
* Correspondence: anna.piras@unipi.it; Tel.: +39-050-2219704
† These authors contributed equally to this work.

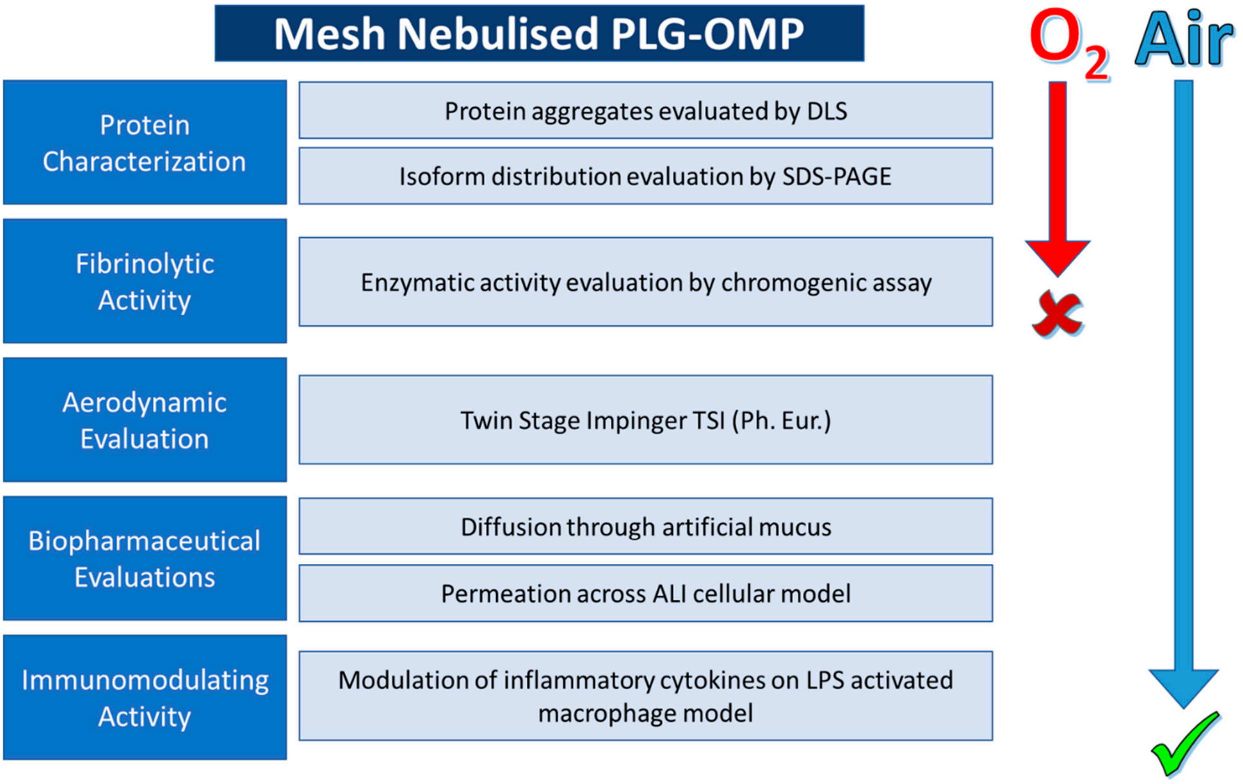

Scheme S1: Workflow of the study. The PLG-OMP eye drop underwent mesh aerosolization either under oxygen or air ambient conditions, simulating clinical off-label administration. The effect of nebulization was assessed on protein features and activity, by collecting the droplets cloud and applying protein characterization assays and proteolytic assay. Due to the observed loss of activity under oxygen administration, only air current conditions underwent to the subsequent investigations.

Protein Size by Dynamic Light Scattering (DLS) and Debye Plot analyses.

PLG-OMP (1 -0.63 mg/ml) underwent to DLS protein analysis. The analysis of the dimensional distribution in intensity shows the presence of two main peaks that persist within all analysed concentrations. The first, corresponding to PLG with an average diameter of 8.4 nm and calculated molecular weight of 93.9 ± 4.2 KDa, in agreement with native plasminogen [1]. The second with an average diameter of 640 nm, corresponding to 0.1% of sample mass, in agreement with product specifications related to the presence of indissoluble aggregates. The sample's progressive dilution causes a change in the relative intensities of the two peaks, favouring the peak at 640 nm, providing indeed a higher scattering intensity, with detriment effect to the main protein scattering peak. Therefore, samples with minor concentration of 0.250 mg/ml could not correctly return PLG MW determination. That concentration was assumed as the lower limit of protein detection.

The data were also confirmed by Debye Plot (Zetasizer Nano series Nano-ZS, Malvern) analysis which returned a molecular weight value in accordance with literature values [1] until 0.25 mg/ml. The Debye Plot was built on the variation of scattering intensity given by the change of protein concentration in solution (0.63-1.0 mg/ml). The average molecular weight was calculated according to: $KC/Ra = 1/M + 2A_2C$ where K is optical constant, Ra is Rayleigh relationship, M is the weighted average molecular weight, A_2 is virial coefficient and C is the concentration of the molecule in solution. Solutions of PLG eye drops were prepared in Milli-Q water and 0.22 μm filtered. The analysis was performed in glass cuvettes at 25 °C, equilibration time of 2 min and 12 interactions of 10s each. Toluene was used as a reference and the differential increase value of refractive index as function of concentration (dn/dc) was setted at 0.185 (average value for protein) [2]. The analysis by Debye Plot led to the correct determination of the PLG molecular weight, obtaining 90.7 ± 1.33 KDa (R^2 0.994), in agreement with literature values [1,3].

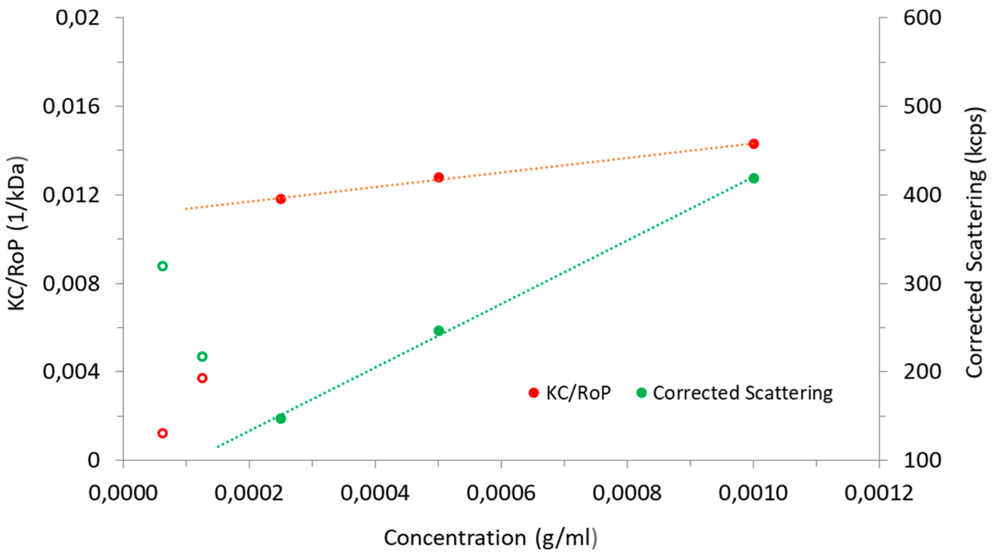

Figure S1. Correlation graph of Debye analysis for the determination of plasminogen molecular weight in PLG-OMP eye drops (0.63-1 mg/ml). The empty points represent concentrations that deviated from linearity (0.63-0.125 mg/ml) and therefore excluded from the analysis.

References

(1) Hayashi, M.; Matsuzaki, Y.; Shimonaka, M. Impact of Plasminogen on an in Vitro Wound Healing Model Based on a Perfusion Cell Culture System. *Mol Cell Biochem* **2009**, *322* (1–2), 1–13. https://doi.org/10.1007/s11010-008-9934-y.

(2) Tumolo, T.; Angnes, L.; Baptista, M. S. Determination of the Refractive Index Increment (Dn/Dc) of Molecule and Macromolecule Solutions by Surface Plasmon Resonance. *Analytical Biochemistry* **2004**, *333* (2), 273–279. https://doi.org/10.1016/j.ab.2004.06.010.

(3) Barlow, G. H.; Summaria, L.; Robbins, K. C. Molecular Weight Studies on Human Plasminogen and Plasmin at the Microgram Level. *Journal of Biological Chemistry* **1969**, *244* (5), 1138–1141. https://doi.org/10.1016/S0021-9258(18)91819-3.

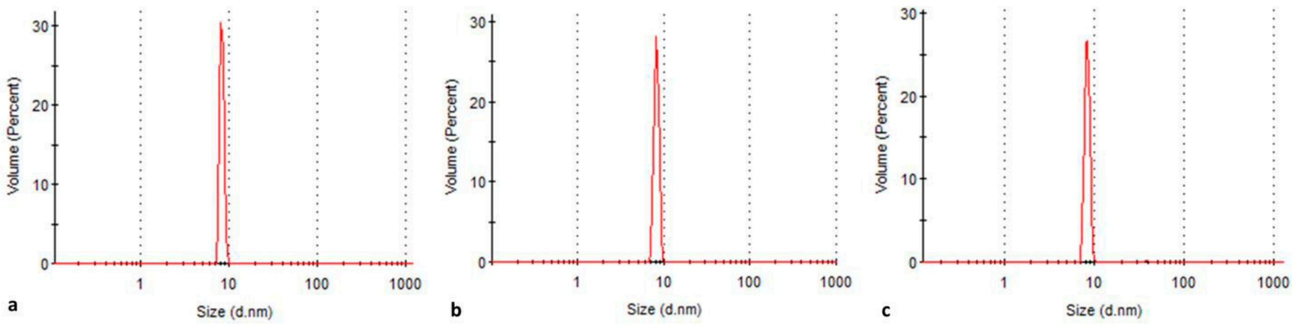

Figure S2. Protein Size distribution by mass in function of scattering intensity data (DLS analysis) of (a) untreated PLG-OMP, (b) collected air-nebulised PLG-OMP (Neb PLG air), and (c) collected oxygen-nebulised PLG-OMP (Neb PLG ox) samples.

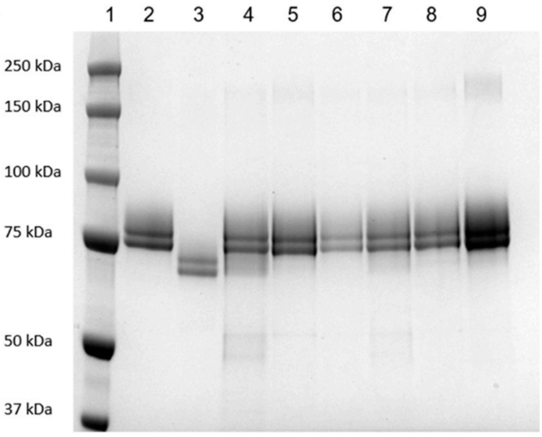

Lane Loading	
1	Mw STD
2	STD Glu PLG
3	STD Lys PLG
4	CTRL
5	Neb PLG air
6	Neb PLG air
7	Neb PLG air
8	Neb PLG air
9	CTRL

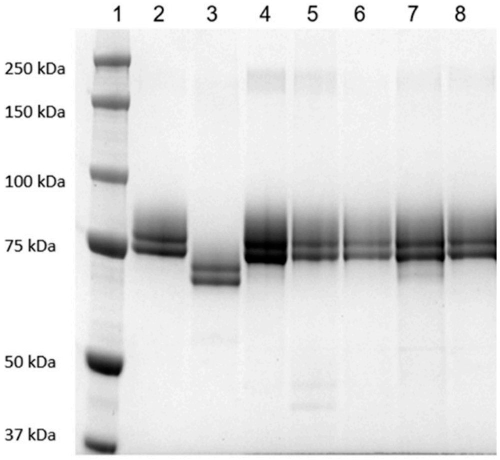

Lane Loading	
1	Mw STD
2	STD Glu PLG
3	STD Lys PLG
4	Neb PLG ox
5	Neb PLG ox
6	Neb PLG ox
7	Neb PLG ox
8	Neb PLG ox

Figure S3. SDS-PAGE assay: nebulised PLG obtained in airflow (Neb PLG air) and in oxygen current (Neb PLG ox) were analysed and compared to untreated PLG-OMP (CTRL) and glycoform standards (STD Glu PLG, STD Lys PLG). Stained gel and lane loading are reported from left to right.

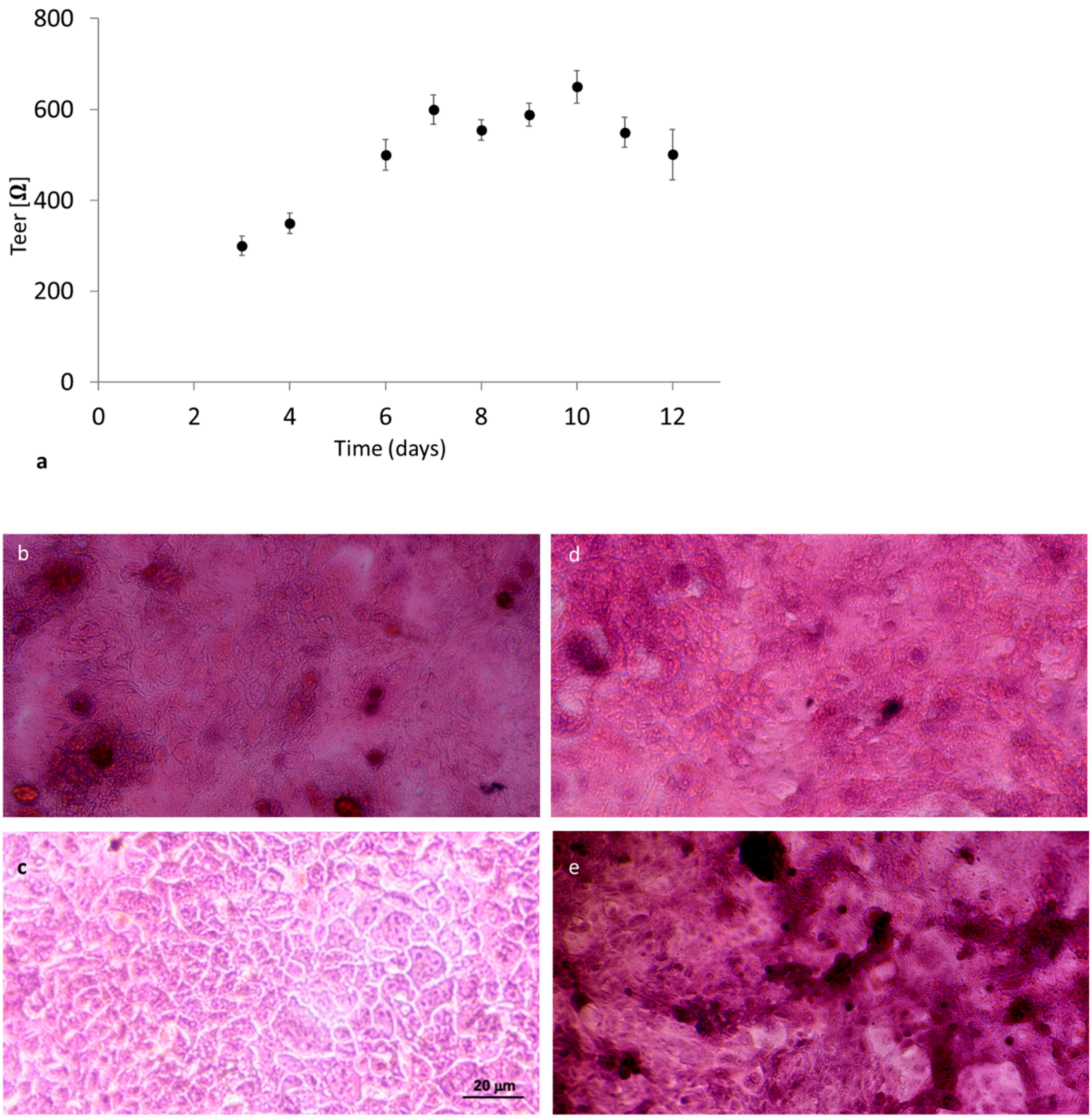

Figure S4. Assessment of NCI-H441 cell monolayers: a) Variation of TEER [Ω] values in NCI-H441 cell ALI-monolayer; b-e) Micrographs of NCI-H441 cell monolayers stained with haematoxylin/eosin at 7(b), 8(c), 10(d), and 12(e) days of culture (10X magnification).

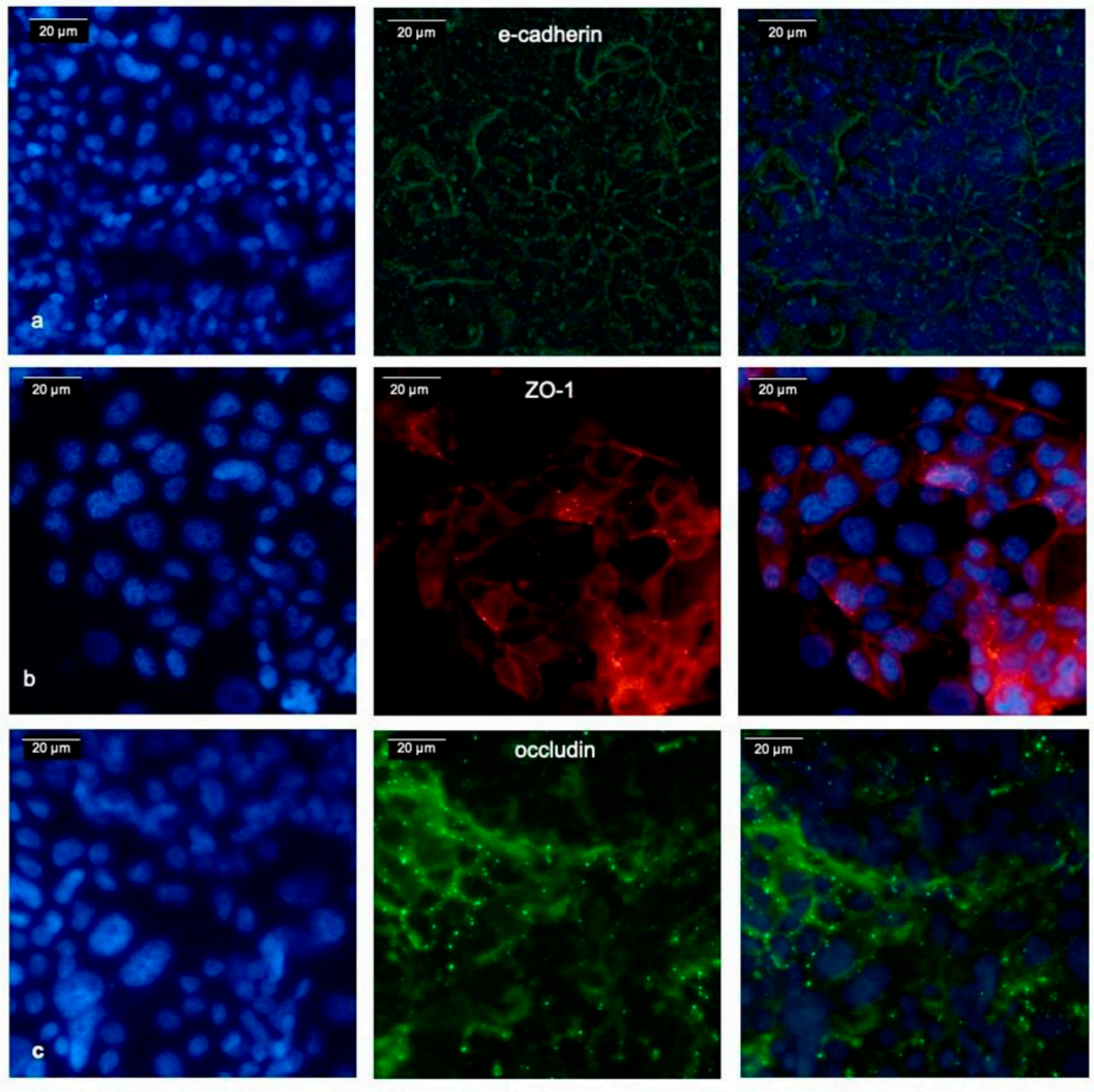

Figure S5. Representative fluorescence micrographs of NCI-H441 cell ALI monolayers grown on Transwell® filters at 8 days of culture (20X magnification). Line a) Single acquisitions of the channels: in blue for the cell nuclei labelled with DAPI, in green for the E-cadherin anchoring junction and merged with the cell nuclei; Line b) Single acquisitions of the channels: in blue for the cell nuclei labelled with DAPI, in red for the tight junction ZO-1 and merge with the cell nuclei; Line c) single acquisitions of the channels: in blue for the cell nuclei labelled with DAPI, in green for the tight junction Occludin and merge with the cell nuclei.

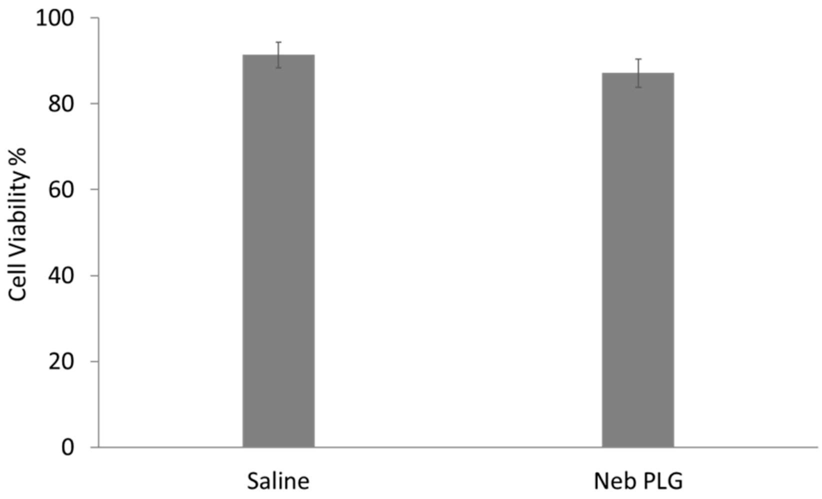

Figure S6. Histogram representing cell viability of NCI-H441 cell monolayer, post nebulisation. PLG-OMP was nebulised for 30 seconds directly on the apical portion of the NCI-H441 monolayers (n=3) and incubated for 2 hours; 0.9% saline solution was used as negative control.

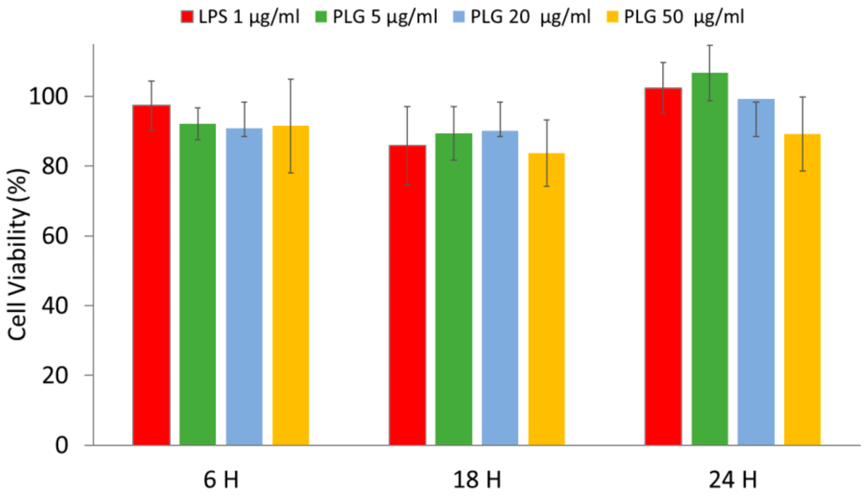

Figure S7. Cell viability study performed on RAW 264.7 cell line at 6, 18 and 24 h, exposed to 5-20-50 µg/ml of PLG-OMP and LPS 1 µg/ml.

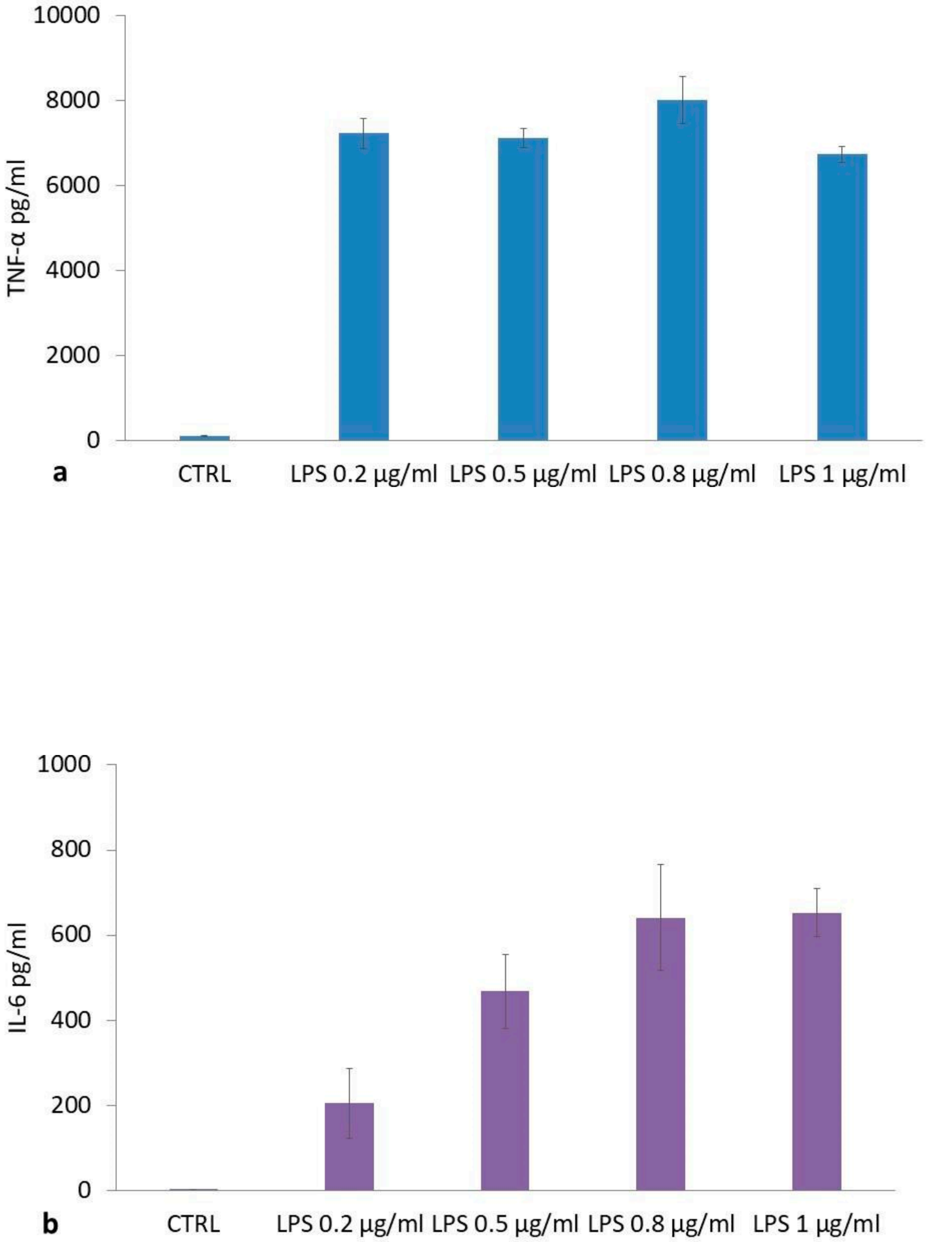

Figure S8. Cytokines production by RAW 264.7 cell line stimulated with different concentrations of LPS (0.2, 0.5, 0.8 and 1 µg/ml). Production of (a) TNF-α and (b) IL-6 after 24 h incubation. Data are reported as mean ± SD of four independent experiments. Untreated cells were used as control.
